# Supplementary material for: Rich complex behaviour of self-assembled nanoparticles far from equilibrium
Source: Nat Commun. 2017 Apr 26;8:14942. doi: 10.1038/ncomms14942 (PMC5414064; doi:10.1038/ncomms14942)
Supplement: Supplementary Information — Supplementary Figures, Supplementary Methods and Supplementary References [file ncomms14942-s1.pdf]

Supplementary Method 1. Custom developed laser system:

The main features of the experimental setup are briefly described below.

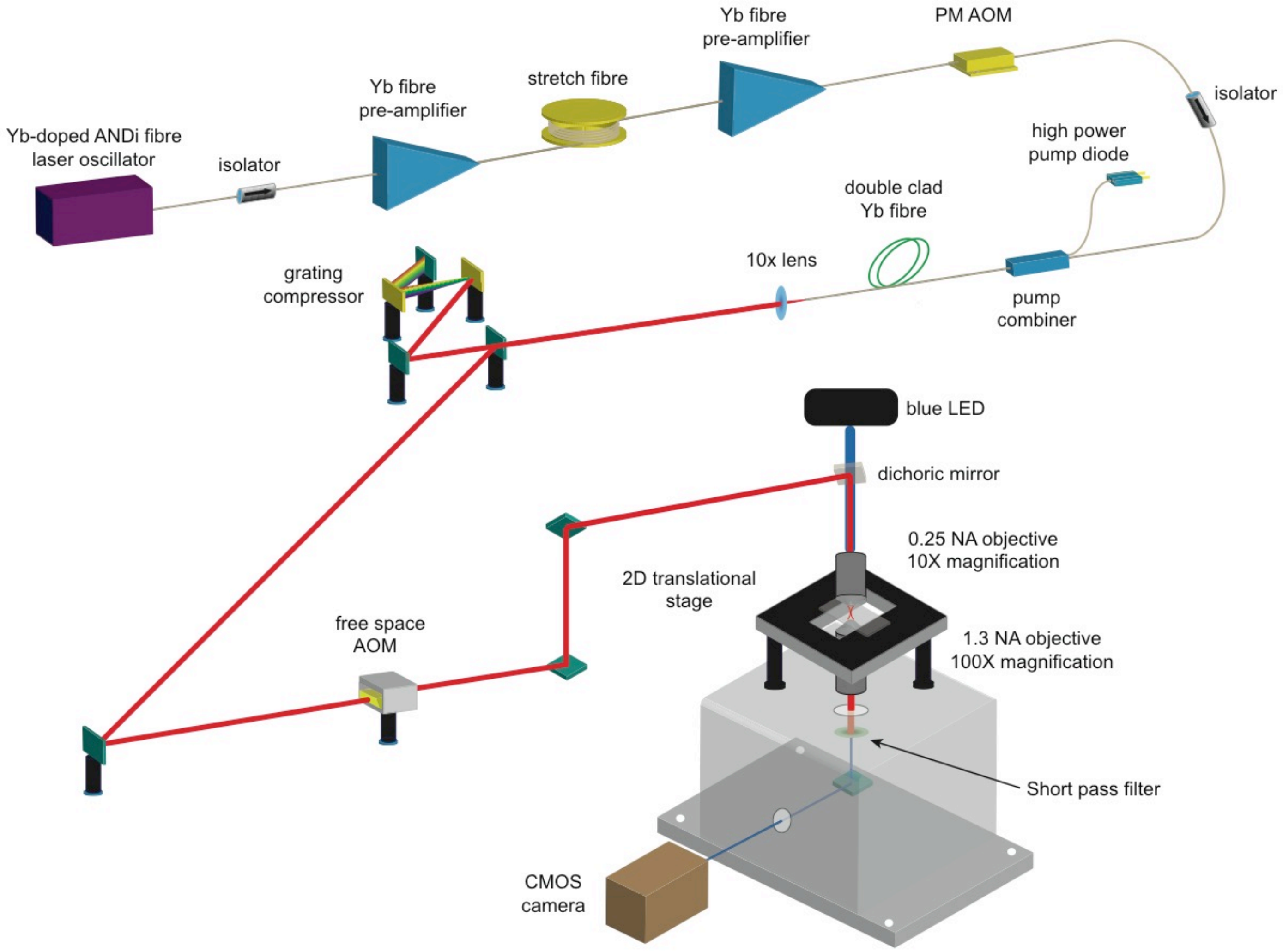

Supplementary Figure 1. Experimental setup is comprised of a customised amplified fibre laser coupled to a computer-controlled 3-axis motorized stage, and a diffraction-limited optical microscope.

Supplementary Method 2. Numerical simulations of the fluid flow:

The velocity field of the fluid flow and the particle trajectories are investigated for different ΔT values, as shown in Supplementary Figure 2. The images show the influence of the temperature difference on the global flow pattern, which preserve their general shape near the bubble. However, the strength of the flow is also important. When ΔT is very low, the drag force cannot overcome the Brownian motion and when it is too high, the drag force overpowers the Brownian motion and pushes all the particles to the edges of the simulated cell. Intermediate values of ΔT provide optimal conditions for self-assembly of nanoparticles at the bubble boundary as shown in the simulated isothermal contours of the fluid flow, where the arrows denote flow trajectories (Supplementary Figure 3a) and as shown through the particle trajectories shown in Supplementary Figure 3b.

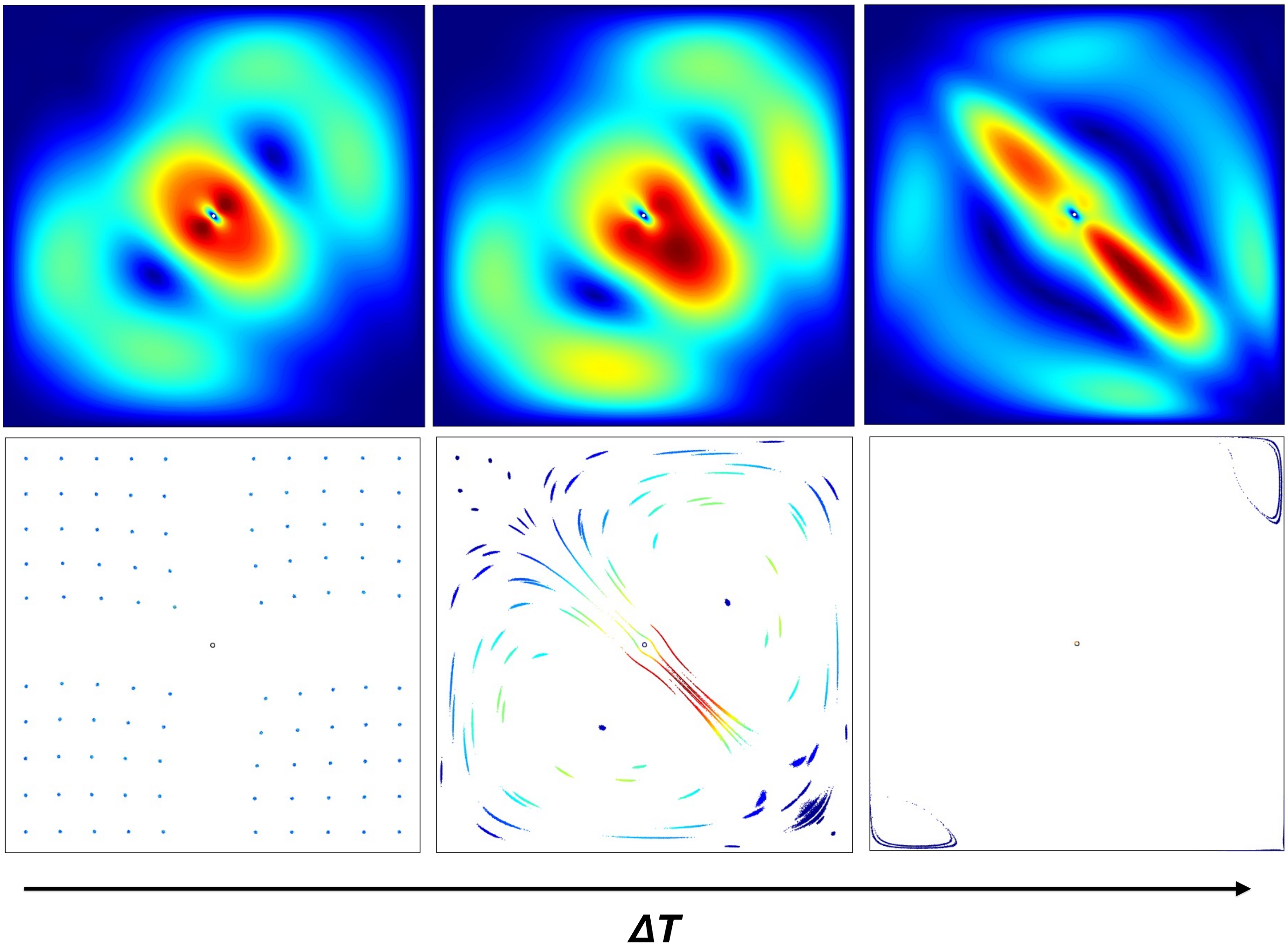

Supplementary Figure 2. Simulations of the fluid flow velocity field for **(a)** low, **(b)** intermediate and **(c)** high values of ΔT, where highest and lowest flow rates are denoted by red and dark blue. Simulations of the particle trajectories for **(d)** low, **(e)** intermediate and **(f)** high values of ΔT, where the fastest and slowest particles are denoted by red and dark blue. The circles in the middle represent the bubble.

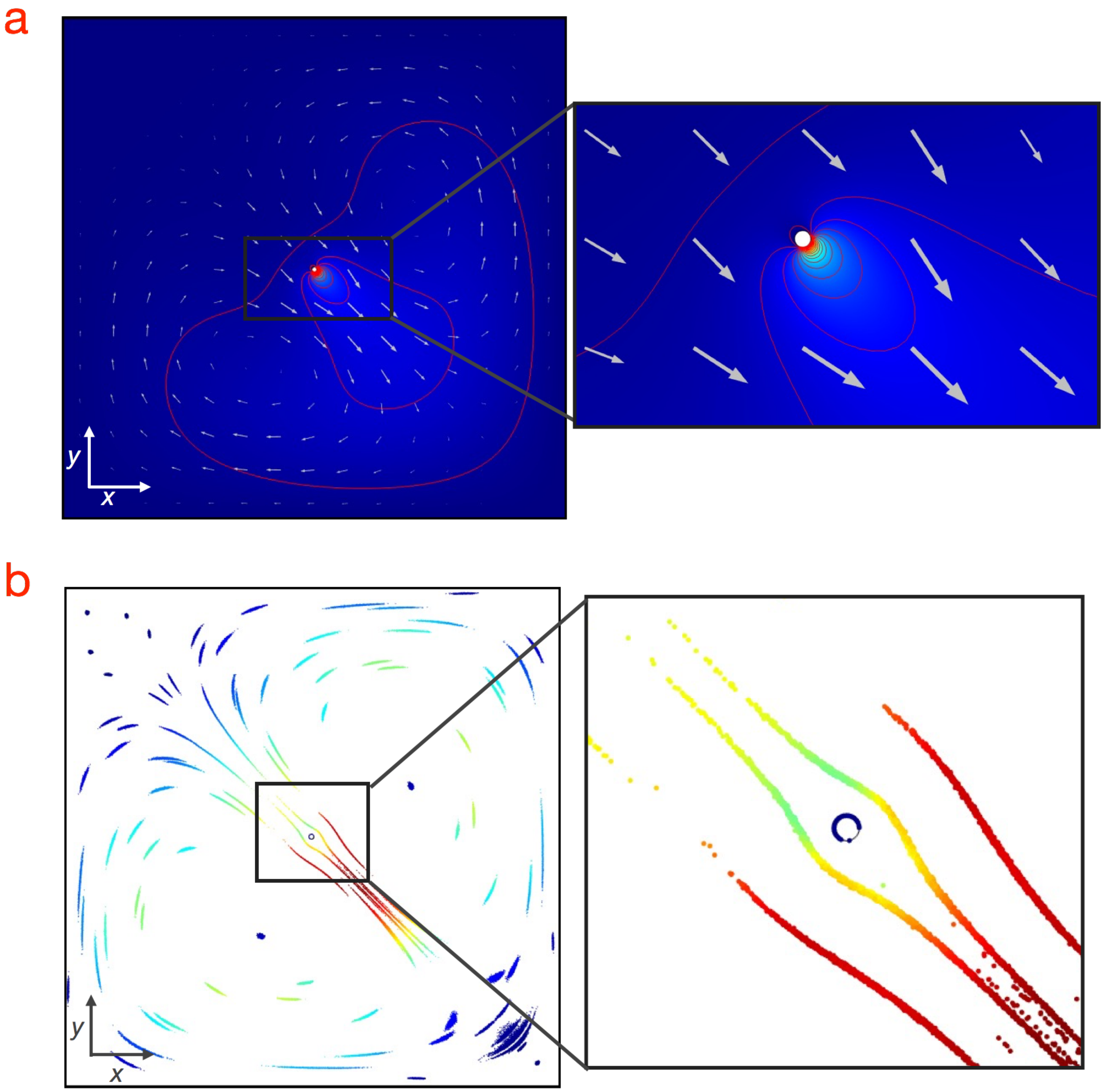

Supplementary Figure 3. Simulations of the **(a)** isothermal contours and **(b)** particle trajectories for the intermediate ΔT value.

Supplementary Figure 4. Typical structure of the phase plane for the dynamic system described by the Toy Model:

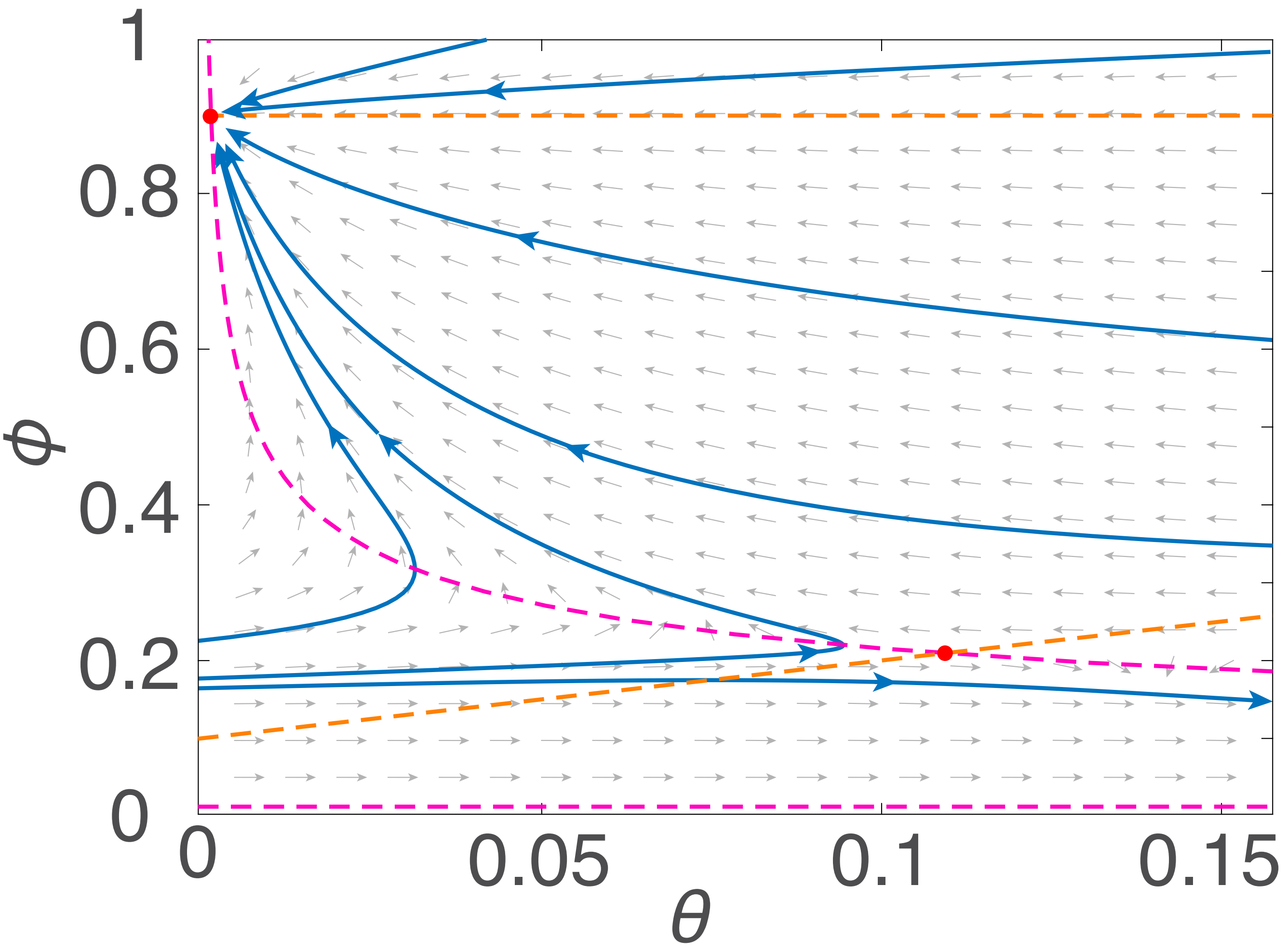

Supplementary Figure 4. Phase plane plot of the aggregation process for $F = 0.001$, $\langle \xi(t) \rangle_{\mathrm{rms}} = 0.1$ and $\tau = 1$. The red dots indicate the fixed points. The upper left one is a nodal sink at (0.14, 0.90) and the central one is a saddle point at (0.49, 0.59). For starting condition of $\phi \lesssim 0.5$, the aggregate is destroyed by the flow and the Brownian motion. For higher initial values of ϕ, achieved with bubble formation, it converges to a dense aggregate.

Supplementary Method 3. Detailed description of data analyses and image processing:

Detailed information on detection algorithms and parameter extraction methods is provided below.

a. Particle detection

We now describe the particle detection procedure. First, digital image pre-processing steps are performed, including intensity level adjustments, 2D order-statistic filtering, and digital eroding. The robustness of the CHT analyses with respect to artefacts, which might be due to optical aberrations, is maintained by finding and selecting the optimum values of the parameters used in pre-processing steps along with the sensitivity and edge thresholding of the CHT procedure. The output of particle detection procedure is a cell array per frame that includes (x, y) coordinates of the particle centres. Supplementary Figure 5 shows the individual steps of the detection procedure.

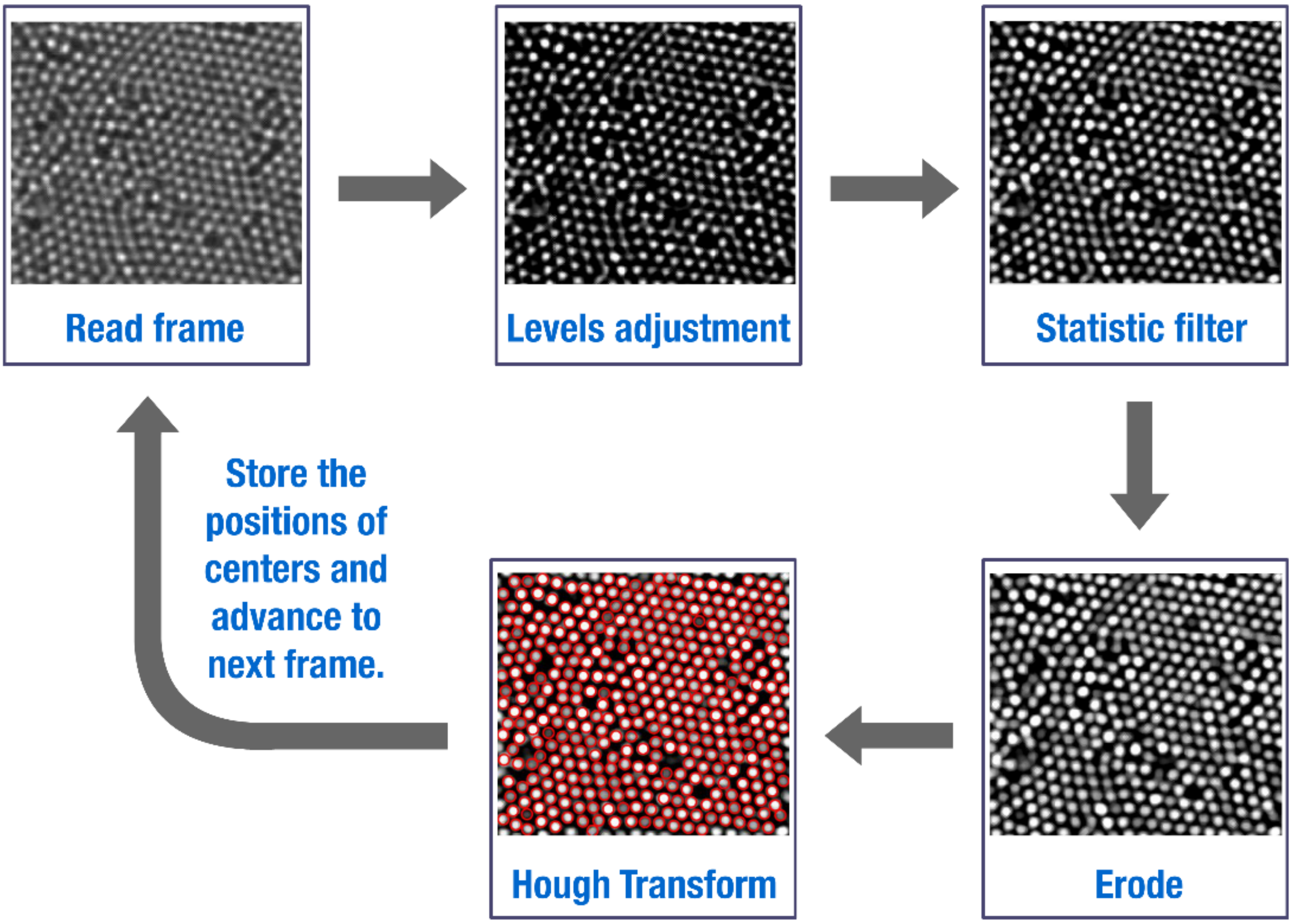

Supplementary Figure 5. Flowchart of the CHT algorithm. Images demonstrate the output of the algorithm at the end of each step. Red circles shown at the Hough transform step are fitted based on central positions the particles.

b. Lindemann parameter calculations

Lindemann parameter can be calculated per particle per frame or it can be averaged over time. Here, we have calculated it per particle as a single scalar value. The algorithm is designed to map each particle according to its relative location with respect to its neighbours. Depending on the relative distances, different phases of materials (solid, liquid or gas) could be distinguished. From solid to gaseous phase the Lindemann parameter changes between 0 and 1, respectively[1,2]. As an example, the blue dots (close to 0) shown in Fig. 2c and 2d of the main text denote the ordered solid structure, whereas green (between 0 and 1) and red (close to 1) dots denote the disordered structure, as in liquid and gaseous phases.

The Lindemann parameter, per particle per frame, is given by the following equation[3]:

$$L_i = \frac{1}{N-1}\sum_{j\neq i}^{N}\frac{\sqrt{\langle {r_{ij}}^2\rangle - \langle r_{ij}\rangle^2}}{\langle r_{ij}\rangle}$$

where L_i is the Lindemann parameter of particle i; N is the number of particles within a pre-specified region around the particle i; j is a parameter that scans over all neighbouring particles within that region; $\langle r_{ij}\rangle$ is the average distance between particles i and j.

We used the Lindemann parameter both to recognize lattice symmetries, as well as to identify material phases. Thus, we used "Lindemann histograms" to identify experimentally observed 2D lattices with different symmetries and their uniformities as shown in Supplementary Figure 6. Top row images show experimentally obtained lattices (raw images), images in the middle row are processed images after Lindemann analyses. Colors of the disks in processed images (middle row) are correlated with the histogram bars (bottom row) according to their Lindemann parameter. As expected, the histogram of hexagonal lattice shows a clear shift toward zero (dark blue) with respect to that of square lattice (light blue), which indicates that the particles are closer to each other in hexagonal symmetry. Furthermore, both lattices are highly uniform (most of the particles are equally distanced), as evidenced from the height differences between the bars.

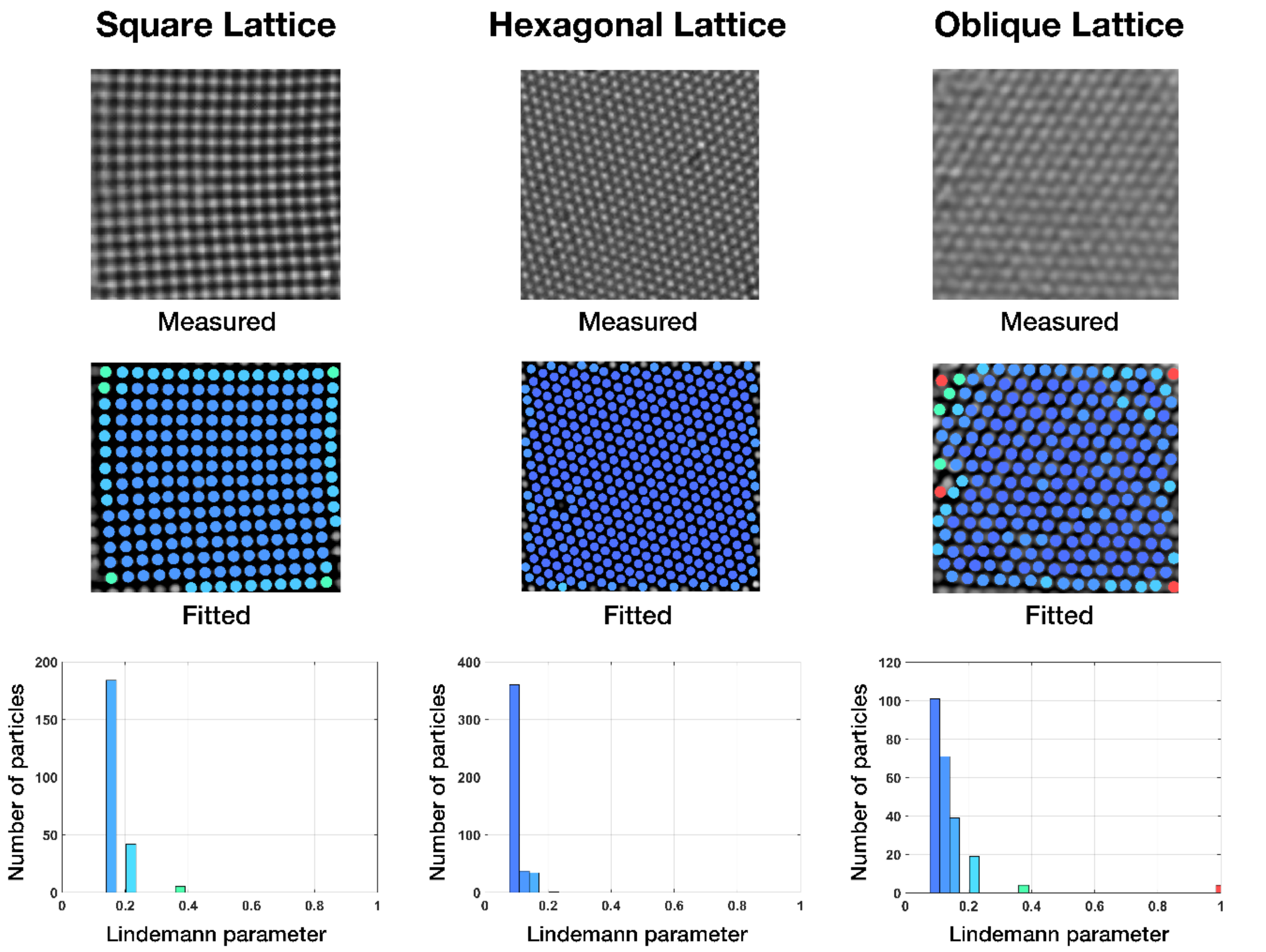

Supplementary Figure 6. Image analyses for hexagonal, square, and oblique lattices. Top row shows experimentally obtained images, middle row shows processed images *via* Lindemann parameter analysis, and bottom row shows the corresponding Lindemann histograms. Cyan- and red-colored data arise from numerical errors (as opposed to a phase change) and can be ignored.

The difference between the hexagonal and oblique symmetries is harder to discern since their Lindemann histograms have the same form. However, we can distinguish them based on the relative heights of the color bars in their respective histograms, which, in principle, can be correlated to directionality: The particles in a hexagonal lattice are equally separated in all directions, as evidenced from the large difference between the three bars shown in its Lindemann histogram. However, in an oblique lattice, the distances are direction-dependent. This also shows up in the Lindemann histogram. In order to independently verify our conclusions based on Lindemann analyses, we also performed pair-correlation and reciprocal lattice analyses for the lattices, which are discussed in the following sections.

c. Giant Number Fluctuations (GNF) calculations

The number fluctuations are calculated following the methodology given in[4], where each frame of the image sequence is split into a number of equal size cells. For each frame, the following quantities are calculated: Total number of the particles divided by the number of cells, N, *i.e.*, the average number of particles per cell, and the standard deviation of the number of particles over the cells, ΔN. If $N_c(i)$ is the number of particles in the ith cell in a frame, then the calculated quantities per frame could be given as

$$N = \langle N_c \rangle$$

$$\Delta N = \sqrt{\langle {N_c}^2 \rangle - \langle N_c \rangle^2},$$

where the averaging is done over cells per frame. The quantities for number fluctuations are calculated for each frame. Then, they are sorted according to values of N and multiple instances with same value of N are averaged, similar to an ensemble average. In case of aggregation, the value of N is expected to serve as indicator of time evolution as well, as both the number of particles and time are growing together until the frame is totally filled up by particles. Scaling factor, α, is used to quantify the number fluctuation, which is defined by

$$\Delta N = N^{\alpha}.$$

If the number fluctuation curve is plotted in logarithmical scale, then α is simply the slope of that curve. Wherever α is close to the value 1, the number fluctuations are considered to be “giant”, and when α value is close to 0.5, the number fluctuations are considered to be “normal”[4].

d. Investigation on Moiré patterns

The experimentally obtained Moiré patterns are reconstructed through image processing: An experimentally obtained image of a hexagonal lattice is superimposed over its 50%-transparent, 18°-rotated (with respect to the vertical axis) replica. As can be seen from the image below, computer-generated and experimentally observed patterns show remarkable agreement[5].

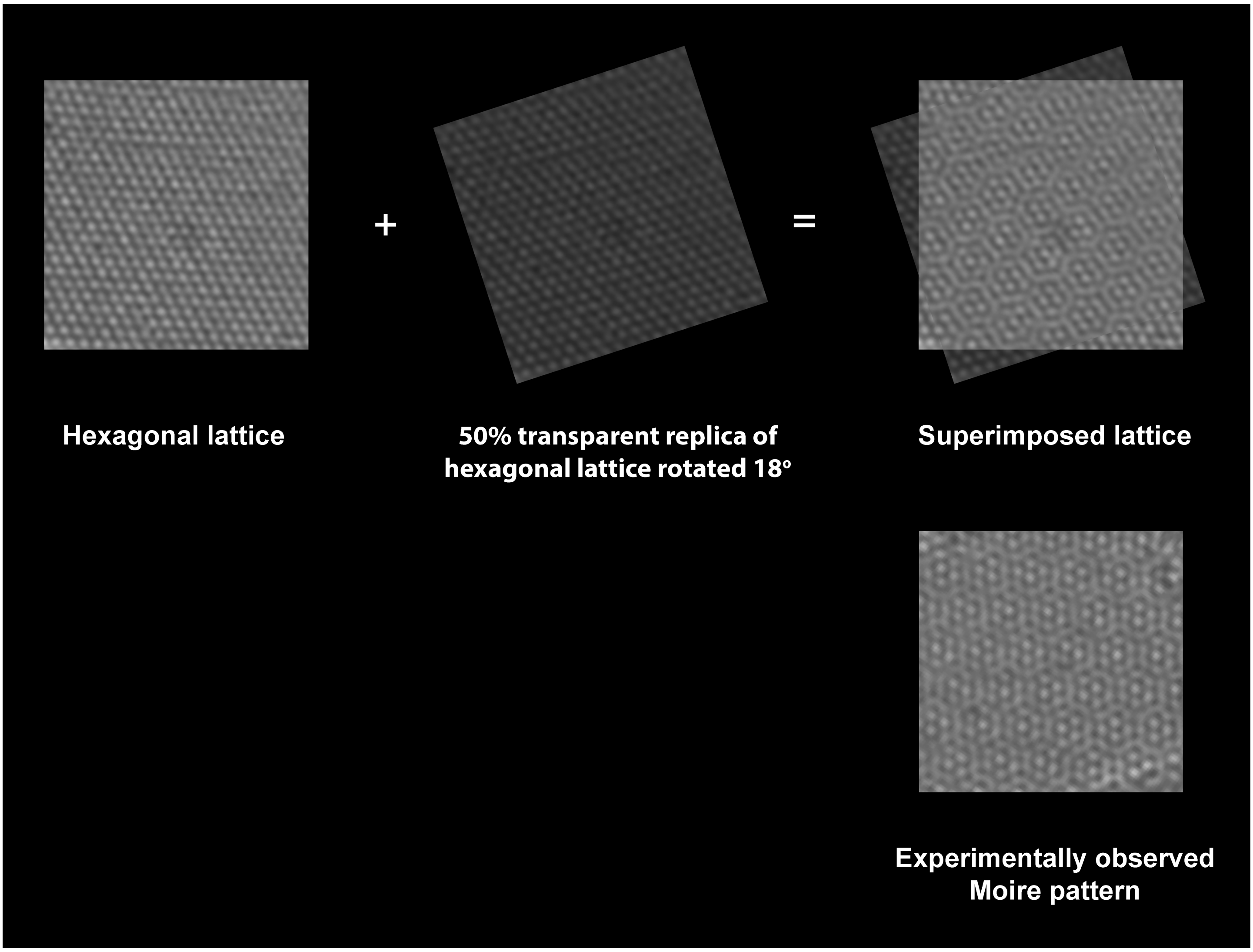

Supplementary Figure 7. Experimentally observed Moiré pattern is simulated through image processing, where a hexagonal lattice is superimposed over its 50%-transparent, 18°-rotated version.

e. Investigation on the multilayer lattices

The initial structure in Fig. 4a is a hexagonal bilayer, where the top and bottom layers of the hexagonal lattice are located on top of each other without any spatial shifting. Therefore, the lattice cannot be in a honeycomb arrangement as described in references[6,7] but should be hexagonal.

In order to prove that, we have performed additional image processing analysis (Supplementary Figure 8): The images have been captured from Movie 6, where the focus of the objective has been intentionally changed (11 s < t < 18 s) to show that we have a bilayer lattice. As can be seen from the movie and from Supplementary Figure 8, upon changing the focus of the objective, the bright circles (Supplementary Figure 8a) turn into dark circles (Supplementary Figure 8b) and they preserve their spatial positions. This means that the bright and dark circles in Layer 1 and 2 are polystyrene particles and they are located on top of each other: (*i*) the red circle in Layer 2 shows a defect, whereas Layer 1 has no defects, which clearly distinguishes one layer from the other (*ii*) by overlapping the two layers (white + white = white, black + white = white, white + black = white, and black + black = black) as shown in Supplementary Figure 8c, we show that there are no dark circles indicating that the layers are in AA stacking, (*iii*) close-up images taken from the same field of view of both layers proves that the particles are located on top of each other (Supplementary Figure 8d).

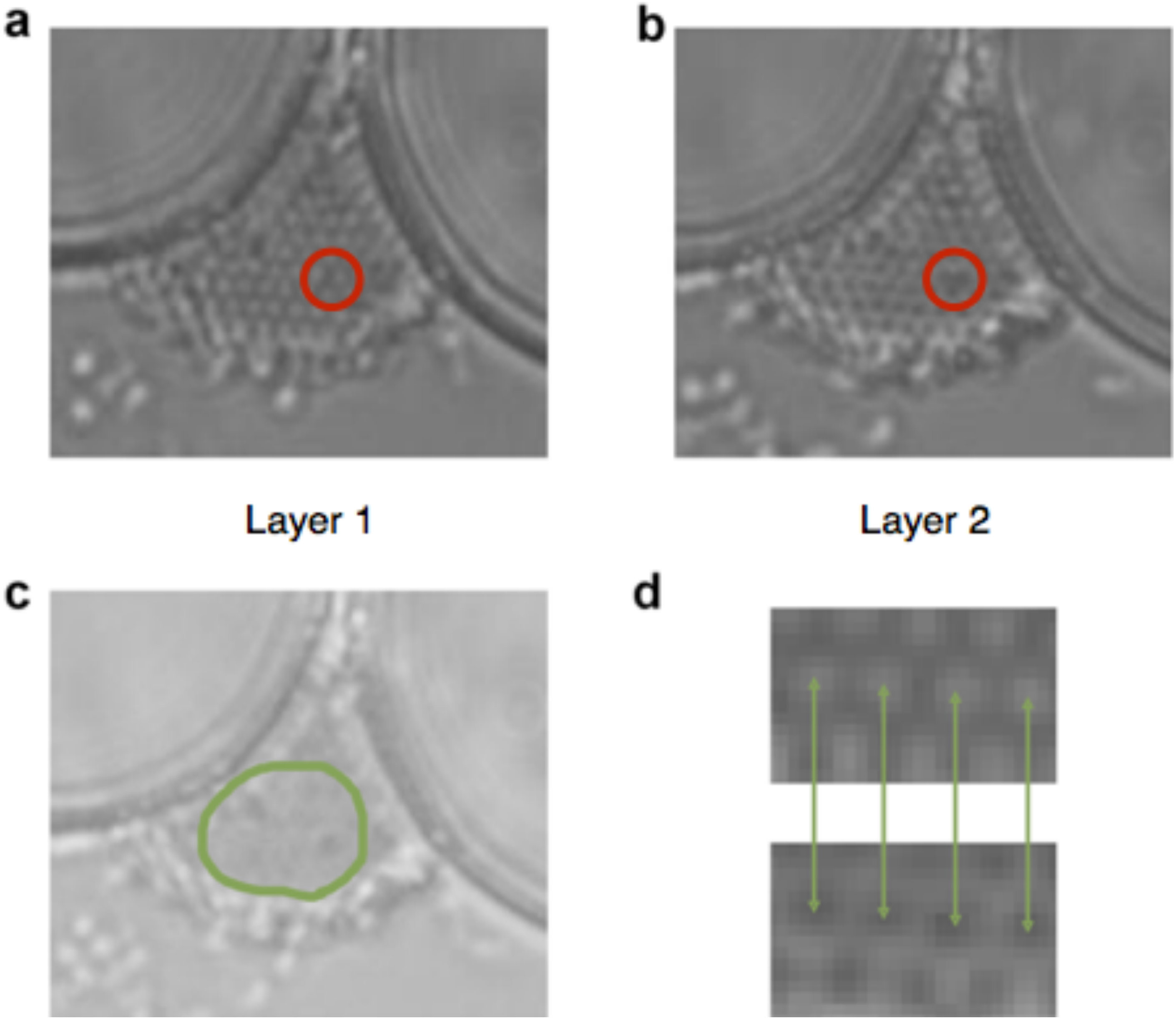

Supplementary Figure 8. Images captured from Supplementary Movie 6 shows polystyrene particles **(a)** as bright circles on the top layer and **(b)** as dark circles on the bottom layer. **(c)** Overlapping image of the top and bottom layers shows that the particles on both layers are located exactly on top of each other. **(d)** Images taken from the same field of view show that the particles on both layers are located on top of each other.

f. Pair correlation function calculations

Pair correlation function can be calculated per frame or it can be averaged over a period of time[8]. Here, we have calculated the function as a single curve per area using

$$g(\vec{r}) = \frac{\mathrm{FFT}^{-1}(|\mathrm{FFT}(I)|^2)}{\rho^2 N(\vec{r})},$$

where FFT is the 2D Fourier transform, I is a binary image created based on the particle positions (*i.e.*, represents each particle as a bright pixel at the centre of the particle), which is padded with zeros to reduce the artefacts originating from Fourier transform, ρ is the average surface density of I, and $N(\vec{r})$ is given by

$$N(\vec{r}) = \mathrm{FFT}^{-1}(|\mathrm{FFT}(W)|^2),$$

where W is the window image of the same size of I with the values 1 over the active (measurement) area of I and 0 outside of it. Supplementary Figure 9 shows pair correlation function analyses of the lattices shown in Supplementary Figure 6.

The distinction in pair correlation plots between the square and hexagonal lattices are clearly seen from the peak positions and shapes. It is difficult to distinguish between the hexagonal and oblique lattices. However, directionality in particle-particle distances causes a shift for oblique lattice.

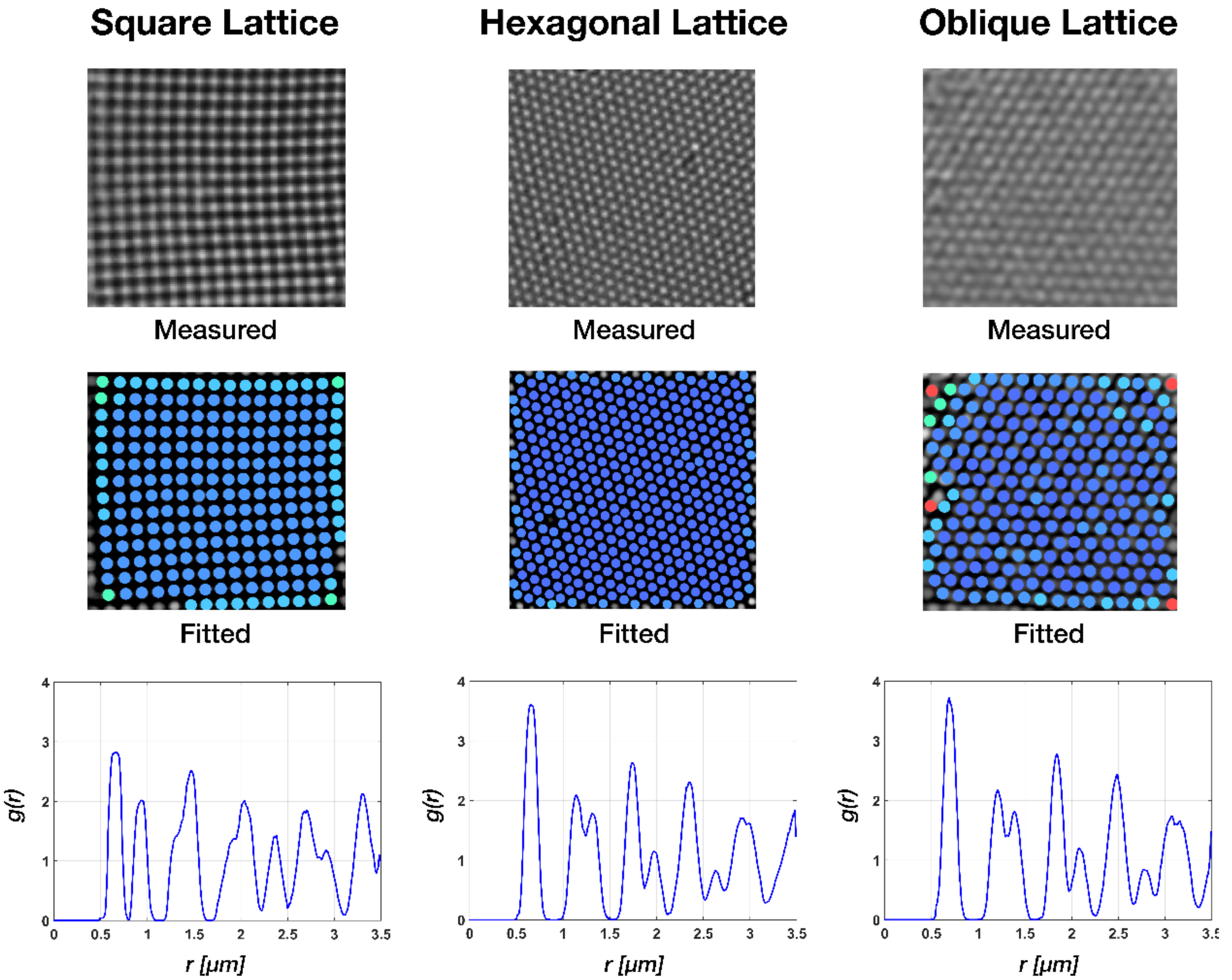

Supplementary Figure 9. Image analyses for hexagonal, square, and oblique lattices. Top row shows experimentally obtained images, middle row shows images processed *via* Lindemann parameter, and bottom row shows the corresponding pair correlation function analyses.

g. Reciprocal lattice calculations

We have calculated reciprocal lattices[9] using experimentally obtained raw images and performed Fourier transform analyses on them as shown in Supplementary Figure 10.

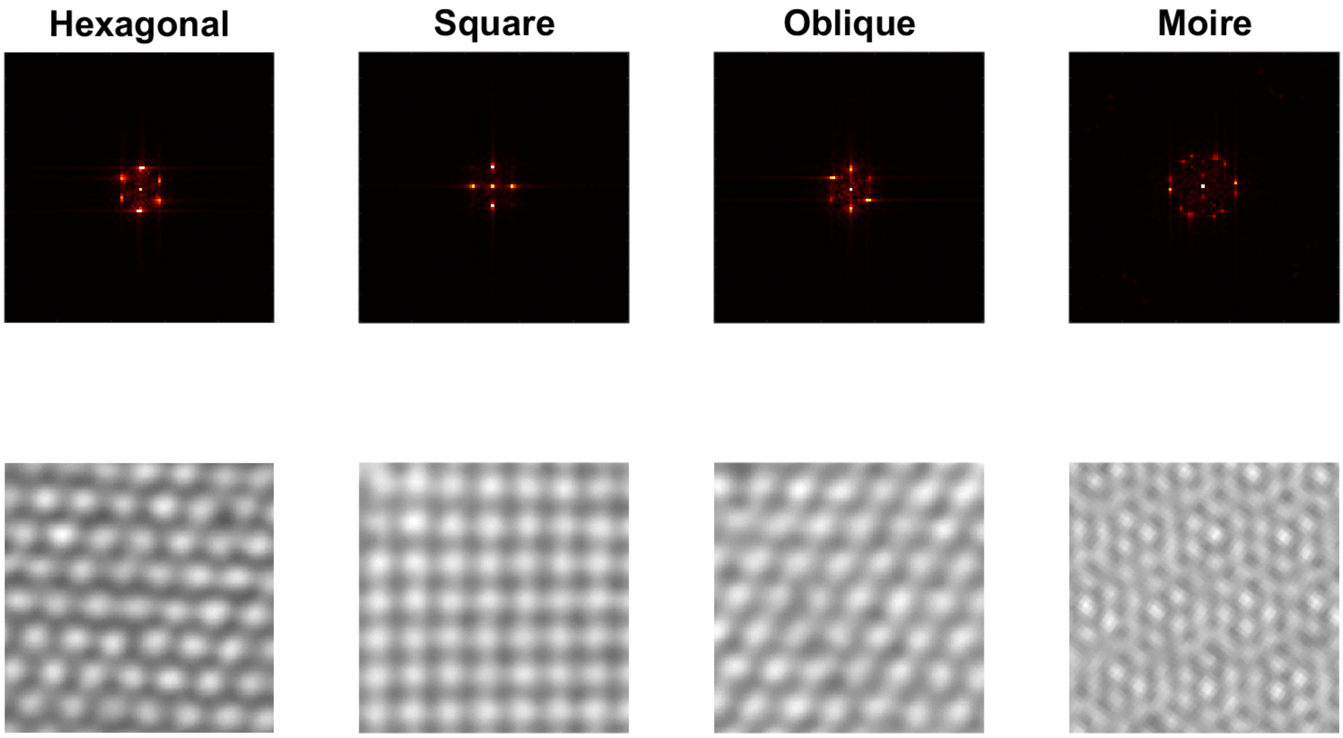

Supplementary Figure 10. Fourier analyses of experimentally observed hexagonal, square, oblique lattices, and Moiré patterns.

Supplementary References

1. Peng, Y. *et. al.* Two-step nucleation mechanism in solid–solid phase transitions. *Nat. Mater.* **14**, 101-108 (2014).
2. Li, Z. H. & Truhlar, D. G. Nanothermodynamics of metal nanoparticles. *Chem. Sci,* **5**, 2605-2624 (2014).
3. Tabti, M., Eddahbi, A., Ouaskit, S. & Elarroum, L. Melting of Argon cluster: Dependence of caloric curves on MD simulation parameters. *WJCMP,* **2**, 139-147 (2012).
4. Palacci, J., Sacanna, S., Steinberg, A. P., Pine, D. J. & Chaikin, P. M. Living crystals of light-activated colloidal surfers. *Science,* **339**, 936-940 (2013).
5. Woods, C. R. *et. al*. Commensurate–incommensurate transition in graphene on hexagonal boron nitride. *Nat. Phys.* **10**, 451–456 (2014).
6. Cargnello, M. *et. al.* Substitutional doping in nanocrystal superlattices. *Nature* **524**, 450-454 (2015).
7. Boneschanscher M. P. *et al.* Long-range orientation and atomic attachment of nanocrystals in 2D honeycomb superlattices. *Science* **344**, 1377-1380 (2014).
8. Veatch, S. L., Machta, B. B., Shelby, S. A., Chiang, E. N., Holowka, D. A. & Baird, B. A. Correlation functions quantify super-resolution images and estimate apparent clustering due to over-counting. *PLoS ONE,* **7**, (2012).
9. Thompson, D. The reciprocal lattice as the Fourier transform of the direct lattice. *Am. J. Phys.,* **64**, 333-334 (1996).
